# Purified hepatitis B virus induces human Mesangial cell proliferation and extracellular matrix expression *In Vitro*

**DOI:** 10.1186/1743-422X-10-300

**Published:** 2013-10-04

**Authors:** Zongli Diao, Jiaxiang Ding, Chenghong Yin, Liyan Wang, Wenhu Liu

**Affiliations:** 1 Department of Nephrology, Beijing Friendship Hospital, Capital Medical University, 95 Yong-An Road, Beijing 100050, China; 2 Department of Infectious Disease, Beijing Friendship Hospital, Capital Medical University, 95 Yong-An Road, Beijing 100050, China

**Keywords:** Hepatitis B virus, Human mesangial cells, Proliferation, Type IV collagen, Fibronectin

## Abstract

**Background:**

Hepatitis B virus (HBV) induces proliferation of human mesangial cells (HMCs), and extracellular matrix expression through the deposition of immune complexes in renal tissue. However, it is unclear whether HBV can directly affect HMCs. In this study, the effects of purified HBV on HMC proliferation and extracellular matrix expression *in vitro* was determined.

**Findings:**

HBV was purified using sucrose density gradient centrifugation. HMCs were co-cultured with purified HBV (10^4^–10^6^ copies/ml) for 48 h, and cell proliferation determined using 5-ethynyl-2′-deoxyuridine immunofluorescence assays. After HMCs were co-cultured with 10^6^ copies/ml purified HBV for 0, 12, 24, 36 and 48 h, expression of type IV collagen and fibronectin was measured using enzyme-linked immunosorbent assays. Three titers of purified HBV (10^4^, 10^5^, and 10^6^ copies/ml) induced HMC proliferation, with the proportion of increases in cell numbers at 24.7 ± 4.3, 31.2 ± 9.4, and 36.8 ± 7.5%, respectively. All these increases were significantly higher than those for the control group (13.6 ± 4.2%) (All *p* < 0.05). Purified HBV (10^6^ copies/ml) significantly increased the levels of type IV collagen and fibronectin in supernatants compared with the control group at 12 and 48 h (All *p* < 0.05).

**Conclusions:**

Purified HBV can directly promote HMC proliferation and expression of type IV collagen and fibronectin, and could be involved in the pathogenesis of HBV-associated glomerulonephritis.

## Introduction

Hepatitis B virus (HBV) infections are prevalent in China [1], and infection with this virus can result in the development of HBV-associated glomerulonephritis (HBV-GN). HBV-GN has become a prevalent type of secondary glomerulonephritis in China [2]. Human mesangial cell (HMC) proliferation and increases in the expression of extracellular matrix proteins, including type IV collagen and fibronectin (FN), were involved in various pathological types of HBV-GN. The most common pathological type of HBV-GN is HBV-associated membranous nephropathy (HBV-MN). Proliferation of HMCs, and increases in extracellular matrix protein levels are more common in HBV-MN compared with the idiopathic form [3]. Additionally, HMC proliferation and increases in extracellular matrix protein expression are common in other pathological types of HBV-GN such as membranoproliferative glomerulonephritis.

The most widely accepted mechanism for increases in HMC proliferation, and expression levels of extracellular matrix proteins in HBV-GN is through the deposition of HBV immune complexes [4]. Many studies have shown that expression of HBV DNA in glomeruli suggests direct viral-induced pathological alterations of HMC could contribute to the pathogenesis of HBV-GN [5,6]. The main effect of HBV DNA in renal tissues on HMCs was because of HBV antigens (HBAg) and HBV antibodies (HBAb) forming immune complexes *in situ*[5,7]. It remains unclear whether HBV can directly affect HMCs; therefore, in this study we purified HBV virons and subviral particles from the serum of HBV-infected patients, and investigated the effects of purified HBV on HMC proliferation, and on the expression levels of type IV collagen and FN.

## Methods

### Isolation and purification of HBV

We isolated HBV from the HBV DNA-positive sera of chronic HBV patients, in our hospital, containing more than 5.0 × 10^8^ copies/ml of circulating HBV. Virus was purified by sucrose density gradient centrifugation as previously described [8].

### Cell culture and cell proliferation assays

HMCs (ScienCell, San Diego, CA, USA) were cultured in Mesangial Cell Medium (MsCM; ScienCell, Carlsbad, USA) at 37°C/5% CO_2_. Cells were seeded into 12-well culture plates at a density of 1.0 × 10^4^ cells/well and incubated for 24 h in RPMI 1640 medium. We then applied HBV at various doses (10^4^, 10^5^, and 10^6^ copies/ml) to HMC cultures. At 48 h post-seeding, cell proliferation was determined using 5-ethynyl-2′-deoxyuridine (EdU) immunofluorescence assays according to the manufacturer’s instructions (Ribobio, Guangzhou, Guangdong, China). The culture medium was replaced with normal MsCM containing 50 μM EdU. After a 3-h incubation, cells were fixed with formaldehyde. After washing with phosphate-buffered saline (PBS) containing 0.5% Triton X-100, cells were washed with PBS and counterstained with Hoechst 33342. Cells were visualized by fluorescence microscopy and using Image-Pro Plus 6.0 (Media Cybernetics, Rockville, MD, USA). The level of HMC proliferation was determined using the following formula:

$$\begin{array}{l} \mathrm{HMC}\;\text{proliferation}=\left(\text{number}\;\mathrm{of}\;\mathrm{EdU}-\text{positive}\;\text{cells}/\text{all}\;\text{cells}\right)\\ \;\times 100. \end{array}$$

### Enzyme-linked immunosorbent assays (ELISAs)

Cells were seeded into 12-well culture plates at a density of 1.0 × 10^4^ cells/well and incubated for 24 h in normal RPMI 1640 medium. We then applied MsCM alone, or 10^6^ copies/ml of HBV in MsCM. At 0, 12, 24, 36, and 48 h post-seeding, the supernatants of each well were collected, and type IV collagen and FN levels measured by ELISA according to the manufacturer’s instructions (Blue-gene, Shanghai, China).

### Statistical analysis

All data were expressed as the mean ± the standard error of the mean (SEM). Statistical analysis was carried out using Tamhane’s T2 test for HMC proliferation, and the Independent-Samples *T*-test for type IV collagen and FN. Differences were considered significant when the *P*-value was less than 0.05. All results were repeated for three times.

## Results

### HMC Proliferation

**Figure 1:**
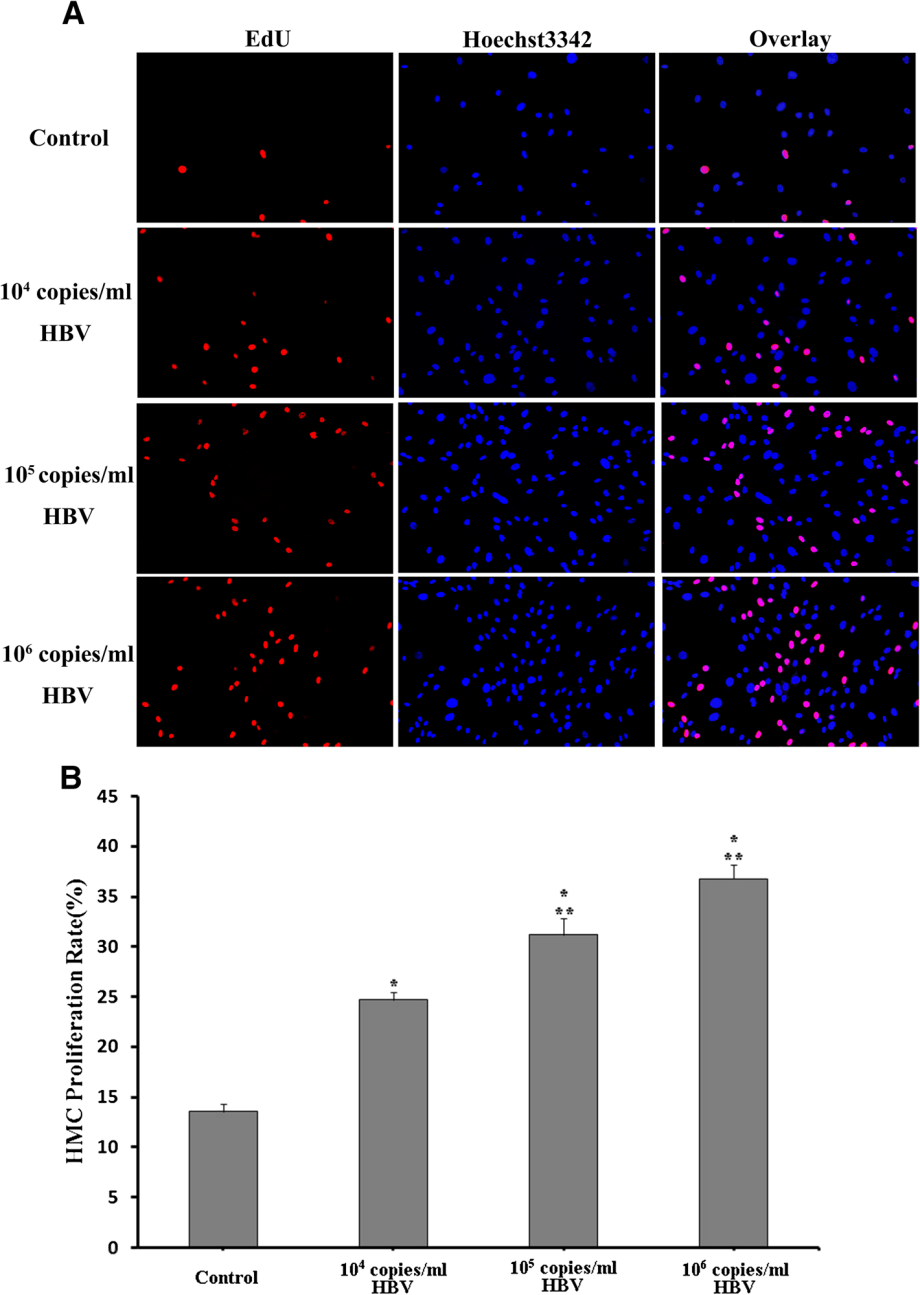
**Various titers of HBV can induce HMC proliferation after 48 h. (A)** Detection of EdU (left) and Hoechst3342 (center) incorporation in cultured HMCs, and overlay images (right). **(B)** HMC proliferation in control and HBV co-culture groups. ^**^p < 0.05 compared with that in normal MsCM. ^**^p < 0.05 compared with that in MsCM containing 10^4^ copies/ml HBV. Results are presented as means ± SEM.

Proliferation of HMCs in control, 10^4^, 10^5^, and 10^6^ copies/ml HBV-infected cultures were increased by 13.6 ± 4.1, 24.7 ± 4.3, 31.2 ± 9.4, and 36.8 ± 7.5%, respectively (Figure 1).

### Extracellular matrix protein levels

**Figure 2:**
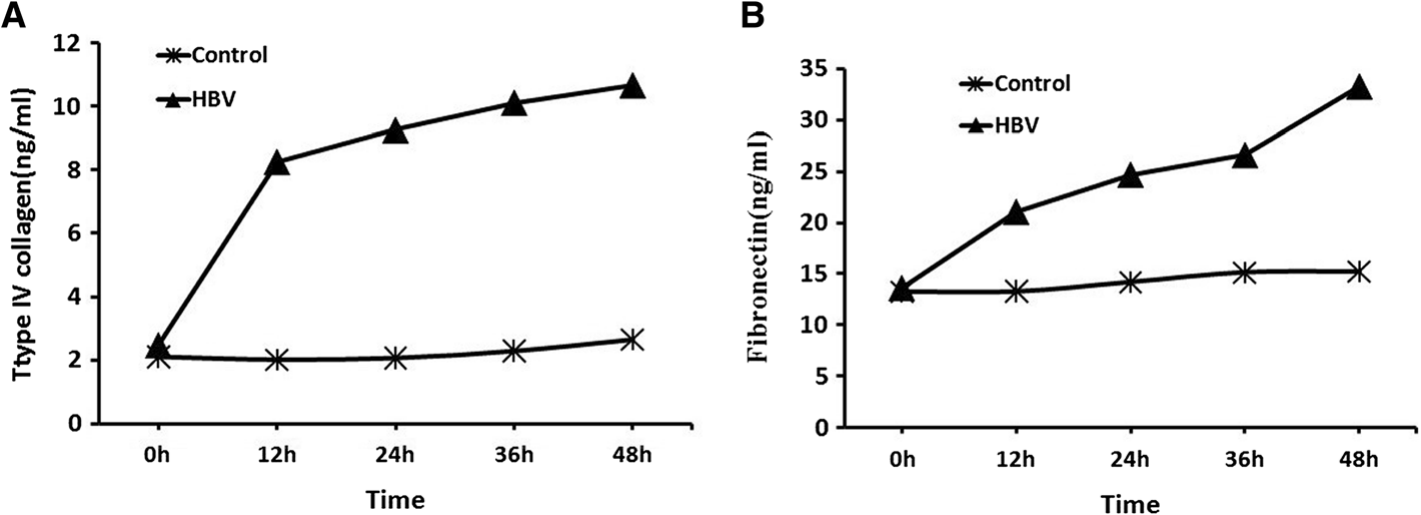
**HMCs were co-cultured with purified HBV.** Type IV collagen **(A)** and FN **(B)** levels in supernatants increased significantly compared with controls at 12, 24, 36, and 48 h (*p* < 0.05).

Using 10^6^ copies/ml HBV significantly increased the expression levels of type IV collagen and FN in HMCs (*p* < 0.05; Figure 2).

## Discussion

In our study, we found that purified HBV significantly increased HMC proliferation, and also increased expression levels of type IV collagen and FN. The major mechanism of increased HMC proliferation and extracellular matrix expression in HBV-GN is suggested to be due to HBV immune complex deposition [6,9]. However, many studies have detected HBV DNA, as both free and integrated forms, in mesangial cells. He et al. have reported that the expression of HBV DNA in the nucleus and cytoplasm of HMCs in HBV-GN patients verified the presence of HBV DNA in HMC [5]. Wang et al. showed that HBV DNA as the integrated form was detected in 82% (41/50) of cases in HMCs by *in situ* hybridization [7]. These observations suggest that HBV DNA can infect HMCs, and that it can directly induce pathological alterations in HMCs that might be involved in HBV-GN [10]. However, these studies were all conducted in renal tissues, and the effects of HBV on HMCs were suggested to be a result of immune complex formation *in situ*[7,11]. Because of confounding factors involving immune complexes that are circulating, it was difficult to show whether HBV can directly affect HMCs in these studies. In our study, we purified HBV from the sera of HBV-infected patients. The HMCs were co-cultured with purified HBV *in vitro*, thereby overcoming the confounding factors related to the immune complex, and we showed that purified HBV directly affected HMCs.

There were some limitations to our study. We did not explore the mechanisms through which HBV increased HMC proliferation and expression of extracellular matrix proteins. HBV X Protein (HBx) acts an indirect transcriptional transactivitor to regulate cell proliferation, transdifferentiation and apoptosis [12]. Some studies have shown that HBx can induce mesangial cell proliferation through the upregulation of interleukin-1β and interleukin-6 [13]. However, in these previous studies, HBx was transfected into mesangial cells artificially; therefore, it remains unknown whether HBV can induce HMC proliferation through a natural infection. Further studies will be required to clarify this.

In conclusion, our findings support the hypothesis that HBV exerts direct effects on HMCs, and will hopefully enrich our understanding of the pathogenesis of HBV-GN.

## Abbreviations

EdU: 5-ethynyl-2′-deoxyuridine; ELISA: Enzyme-linked immunosorbent assay; FN: Fibronectin; HBV: Hepatitis B virus; HBV-GN: HBV-associated glomerulonephritis; HBV-MN: HBV-associated membranous nephropathy; HBx: HBV X protein; HMC: Human mesangial cells; MsCM: Mesangial cell medium.

## Competing interests

The authors declare that they have no competing interests.

## Authors’ contributions

DZ and JD carried out all experiments in this study, collated the information, performed the literature search and drafted the manuscript. CY performed the EdU assays. LW performed the ELISA experiments. WL, the corresponding author, designed the research project, performed the literature search and prepared the manuscript. All authors read and approved the final manuscript.
